# The macroeconomic burden of noncommunicable diseases associated with air pollution in China

**DOI:** 10.1371/journal.pone.0215663

**Published:** 2019-04-18

**Authors:** Simiao Chen, David E. Bloom

**Affiliations:** 1 Heidelberg Institute of Global Health (HIGH), Faculty of Medicine and University Hospital, Heidelberg University, Heidelberg, Germany; 2 Department of Global Health and Population, Harvard T.H. Chan School of Public Health, Boston, MA, United States of America; The Ohio State University, UNITED STATES

## Abstract

**Background:**

While a few studies have tried to estimate the economic burden of noncommunicable diseases (NCDs) associated with air pollution, most previous studies have methodological limitations. For example, neither the cost of illness approach nor the value of a statistical life approach accounts for economic adjustment mechanisms (i.e., they do not include substitution of labor lost due to an illness with capital or other workers), and neither approach considers disease impact on physical and human capital. Furthermore, since new evidence shows that air pollution is also linked to diabetes, previous studies did not estimate the economic costs of diabetes associated with air pollution. The total economic costs of NCDs associated with air pollution under a comprehensive framework therefore remained unexplored.

**Objectives:**

This study uses a human capital–augmented production function framework to analyze and estimate the macroeconomic impact of NCDs associated with air pollution in China in 1990–2030 and in 2015–2030. It makes several contributions—beyond those of the extant literature—to understanding the economic burden of NCDs associated with air pollution. It does this by accounting for economic adjustment mechanisms and by incorporating human capital into the model.

**Methods:**

In our framework, aggregate output is produced according to a human capital–augmented production function that accounts for the effects of projected disease prevalence. NCDs associated with air pollution affect the aggregate output through three pathways: 1) Mortality effect—when working-age individuals die from a disease, aggregate output decreases because physical capital is an imperfect substitute for the loss of human capital in the production process. 2) Morbidity effect—when working-age individuals suffer from a disease but do not die from it, their contribution to overall output also decreases depending on disease severity; for example, they might work fewer hours or with lower productivity, or they might retire earlier. We also incorporate age-specific human capital to account for education-related productivity differences between members of different cohorts who are differentially affected by NCDs. 3) Treatment cost effect—when households in which members suffer from a disease use part of their savings to cover the out-of-pocket share of their treatment costs, physical capital accumulation diminishes. Our estimates are based on the recently updated Global Burden of Disease epidemiology data, which identify four pathways through which air pollution affects health: cardiovascular diseases, respiratory diseases, cancer, and diabetes.

**Results:**

Total losses from NCDs associated with air pollution in China in 1990–2030 are estimated to be $1,137 billion (constant 2010 USD) and in 2015–2030 are estimated to be $499 billion (constant 2010 USD). Cardiovascular diseases account for the highest burden, followed by chronic respiratory diseases, diabetes, and cancer. Treatment costs account for nearly 30% of the total economic burden of NCDs associated with air pollution. We also find that the share of economic burden associated with treatment costs is highest for diabetes. This is mainly driven by the fact that, on a per case basis, diabetes has a lower health burden than other diseases associated with air pollution.

**Discussion:**

The NCDs associated with air pollution impose a large economic burden on China.

## Introduction

In recent years, severe air pollution has plagued China as its economy and its citizenry reckon with the dual environmental challenges of tradition and modernization. Though many locations in industrialized countries have also suffered from air pollution problems—for example, the Meuse Valley in Belgium in 1930 [1], Pennsylvania in 1948 [2], and London in 1952 [3]—China’s situation is extraordinary because of the number of people affected, the air pollution’s intensity, and the geographical impact. Air pollution has become one of China’s most pressing public health concerns as the number of extremely polluted days has risen substantially [4]. While the World Health Organization (WHO) recommends an annual particulate matter concentration of 20 μg/m^3^ [5], studies suggest that the annual mean particulate matter concentration in China was five times that level in 2006–2010 [6]. Reuters reports that by the end of 2016, the concentrations of airborne pollutants in major northern Chinese cities surpassed the WHO guideline by a factor of 100 [7].

The Institute for Health Metrics and Evaluation divides air pollution into three categories: 1) ambient ozone pollution, 2) ambient particulate matter pollution, and 3) household air pollution from solid fuels. Sources of air quality deterioration include motor vehicle emissions, power generation, smelting and metal processing, industrial combustion, and others. In China, the dominant pollutants are gaseous contaminants and fine particles, of which PM2.5 (particulate matter of diameter up to 2.5 μm) is the most crucial component [8]. In Beijing, the peak hourly concentration of PM2.5 (800 μg/m^3^) is more than 32 times higher than the WHO recommended level (25 μg/m^3^) [9].

Awareness of the health consequences of air pollution is growing. Studies suggest that air pollution in China is positively associated with mortality and morbidity rates of noncommunicable diseases (NCDs). For example, evidence indicates that air pollution is associated with respiratory mortality [6], coronary heart disease mortality [10], and ischemic heart diseases and cerebrovascular diseases [11]. Recent evidence shows that air pollution is also likely to be associated with kidney diseases [12] and diabetes [13]. The health impact varies greatly from region to region [14].

NCDs associated with air pollution can affect economic growth in many ways. For example, because more air pollution may be linked to unhealthier populations, people’s productivity may decrease because they take more sick days and are less energetic and productive [15, 16]. Recent studies show the negative causal effect of short-run pollution exposure on hours worked [17, 18] and productivity while at work [19–21]. An unhealthier population that suffers from more air pollution is also less educated because children with poor health have lower school attendance rates and worse cognitive function [22, 23]. For example, studies conducted by economists show a link between air pollution and student absences [24, 25]. In addition, when these children grow up, they have lower incomes than healthy children do. Higher morbidity rates due to air pollution may also lower probability of employment and early retirement, which may reduce labor supply [26–29]. Furthermore, NCD treatment requires a greater investment of resources if air pollution is more severe. For example, evidence shows a causal effect of short-run pollution exposure on medication purchases [30]. Because spending on NCDs reduces government funds for other investments and impedes the accumulation of physical and human capital, reducing or eliminating air pollution would lower NCD incidence, which could allow these resources to be saved and diverted to other important areas, such as education and infrastructure.

Previous studies in China found that the economic impact of air pollution accounts for 0.72−10% of its GDP based on different study settings [31–38]. However, most of the previous studies analyzing the total economic loss due to NCDs associated with air pollution have methodological limitations [10, 39]. For example, neither the cost of illness approach nor the value of a statistical life approach accounts for economic adjustment mechanisms (i.e., they do not include substitution of labor lost due to an illness with capital or other workers), and neither approach considers disease impact on physical and human capital. Furthermore, since new evidence shows that air pollution is also associated with diabetes, previous studies did not estimate the economic costs of diabetes associated with air pollution. The total economic costs of NCDs associated with air pollution under a comprehensive framework therefore remained unexplored.

To overcome the methodological limitations in the previous studies, we use a recently developed macroeconomic model [40, 41] in this paper to answer the following question: if no air pollution were present, how much economic gain could China achieve in 1990–2030 due to lower rates of NCDs? In other words, what is the macroeconomic burden of NCDs associated with air pollution in China in 1990–2030?

In this framework, aggregate output is produced according to a human capital–augmented production function that considers how the impact of projected disease prevalence and NCDs associated with air pollution influences the economy via three pathways: 1) Mortality effect—when working-age individuals die from a disease, aggregate output decreases because physical capital can only partially substitute for the loss of human capital in the production process. 2) Morbidity effect—when working-age individuals suffer from a disease but do not die from it, their contribution to overall output also decreases depending on disease severity. For example, they might work fewer hours or with lower productivity, or they might retire earlier. When calculating mortality and morbidity effects, we also incorporate age-specific human capital to account for education-related productivity variance between members of different cohorts who are differentially affected by NCDs. 3) Treatment cost effect—because households in which members suffer from a disease use part of their savings to cover the out-of-pocket share of their treatment costs, physical capital undergoes a loss. This expenditure negatively affects economy-wide physical capital accumulation and thus is linked to a loss in aggregate output.

## Methodology

### The model

According to the Global Burden of Disease (GBD) Study 2017 [42], air pollution is associated with four disease categories: cardiovascular diseases, chronic respiratory diseases, cancer, and diabetes. In this analysis, we calculate the economic burden for these four disease categories separately and collectively.

To quantify the economic burden associated with a particular disease (or disease category), we need to compare economic performance between the following two scenarios (over the period 1990–2030):

### Status quo scenario

This is the business-as-usual scenario: no intervention that would reduce the mortality rate of a disease further than already projected is implemented.

### Counterfactual scenario

This is the scenario in which we assume complete elimination of disease burden associated with air pollution (with respect to the disease category of interest) at zero cost. Intuitively, this scenario models what would happen if air pollution were no longer associated with certain diseases.

The macroeconomic burden of air pollution (due to the disease category of interest) is then calculated as the undiscounted cumulative difference in projected annual gross domestic product (GDP) between these two scenarios.

**Fig 1:**
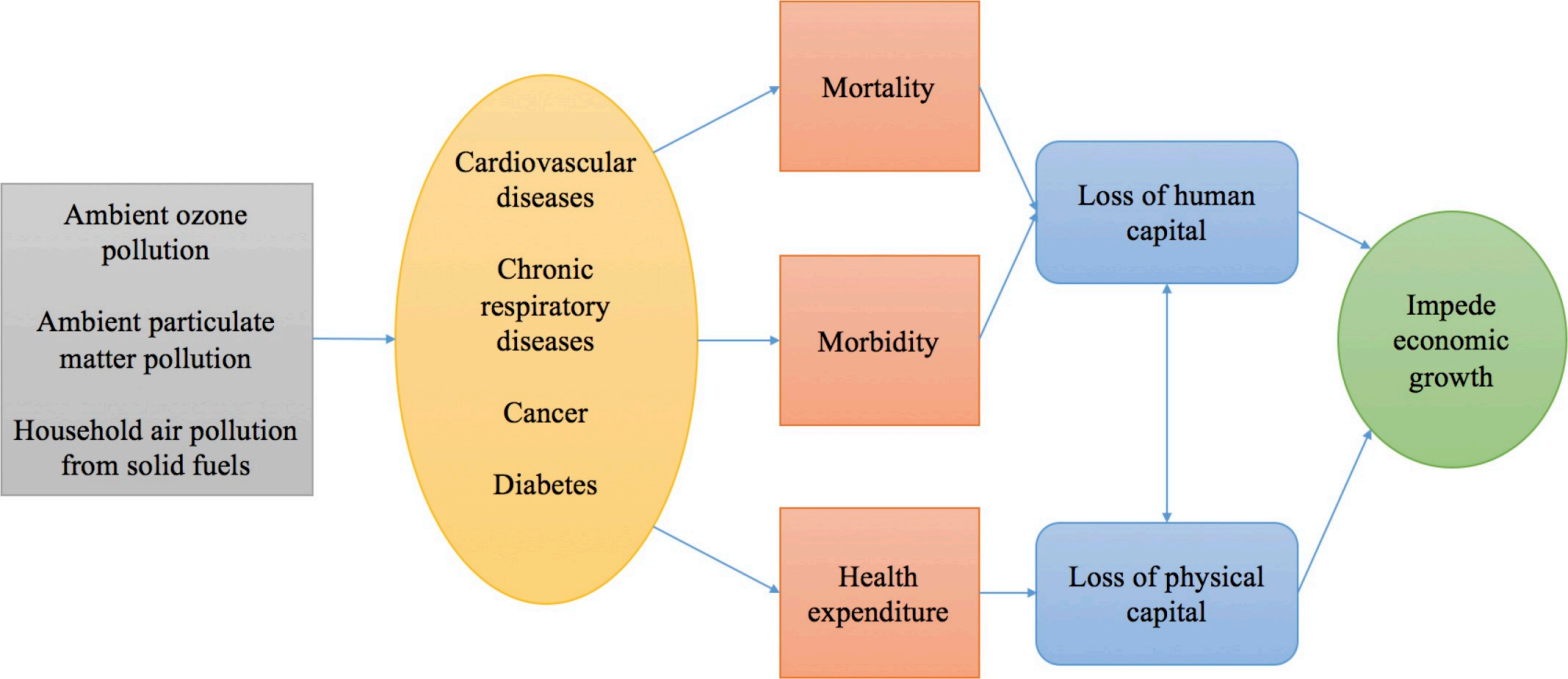
Underlying mechanisms linking NCDs associated with air pollution to economic growth.

To construct economic projections for each scenario, we utilize the model developed by Bloom et al. [40], which allows us to quantify the impact of noncommunicable diseases on aggregate output. The model is based on Lucas’s [43] economic growth model, in which aggregate income growth is assumed to depend on technological progress, capital accumulation, and human capital accumulation. The Lucas model has been widely used to model aggregate output and macroeconomic performance. Fig 1 summarizes the pathways this model addresses. The outcomes of these channels translate into a loss in aggregate output through the Lucas production function and affect the economy’s production possibility frontier over time.

NCDs associated with air pollution can affect the economy via the following pathways: 1) Effective labor supply is reduced due to mortality associated with air pollution; air pollution is associated with higher NCD-induced mortality, which reduces the population and thus the number of working-age individuals. 2) Effective labor supply is reduced due to morbidity associated with air pollution because air pollution may be linked to a higher probability of getting some NCDs that do not directly cause death (e.g., chronic respiratory diseases). These illnesses may reduce working productivity and increase absenteeism. In our framework, we also consider the average human capital level because NCDs associated with air pollution disproportionately affect older age groups. 3) Physical capital accumulation is impeded because savings finance part of the treatment costs. See S1 Appendix for more details.

NCDs’ effects on production potential can be modeled by projecting the age structure of the workforce, its human capital level, and the employment structure and using the projected disease prevalence over the relevant time horizon to calculate production loss. Note that we must differentiate among the disease burdens in different age and education groups, because the mortality and morbidity of people who are not in the labor force (retirees, children, etc.) will not affect aggregate output. In addition, people with different age and education levels will have different experience and productivity and, thus, different human capital. Each year, economic output is estimated according to the previously described production function approach.

### Data sources

We present direct estimates for all NCDs (including cardiovascular diseases, chronic respiratory diseases, cancer, and diabetes) associated with air pollution in China. For diabetes, we obtained the treatment cost information for China from the International Diabetes Federation [44]. For the other three disease categories, we sourced the treatment cost data from other countries [45–47]. For each disease category, we calculate the per case costs for the countries with data and extrapolated costs for China under the assumption that the per case costs are proportional to the per capita health expenditure (from World Bank [48]). This technique has been proposed and used in [49] and [50]. Now we have the disease-specific treatment cost per capita (transformed from per case costs with prevalence data from GBD). We then approximate the treatment cost associated with pollution by assuming that the cost is proportional to DALYs (the data for DALY are from GBD 2017 [42]). For example, if air pollution accounts for 7% of the total DALYs of cancer according to GBD 2017, the respective treatment cost associated with air pollution for cancer will then be calculated as 0.07*total treatment cost of cancer. In order to adjust for rising medical costs, we assume that the per capita treatment costs share the same growth rate as per capita health expenditure. We project per capita health expenditure to 2030 by considering the trend between 2000 and 2015 [48]. Table A in S1 Appendix lists the estimates of treatment costs for NCDs associated with air pollution in China as of 2015 (in constant 2010 USD).

The data on mortality and morbidity associated with air pollution (i.e., years of life lost due to premature mortality [YLLs] and years lost due to disability [YLDs]) for each of the relevant disease categories in China are based on Global Burden of Disease estimates from 1990–2017 [42]. Mortality for 2015–2030 is projected assuming the same annual growth rate of mortality rates as in 2010–2017. We obtain age-specific labor force projections from the International Labour Organization [51] and age-specific data on average years of schooling from the Barro-Lee education database [52]. Data on economic variables are obtained from the World Bank [53, 54] and the Penn World Table (2017) [55]. We use other necessary parameters for calculating human capital from the literature [56]. See Table B in S1 Appendix for a detailed description of the parameter values and data sources used in the macroeconomic model.

## Results

### Health burden of NCDs associated with air pollution

**Fig 2:**
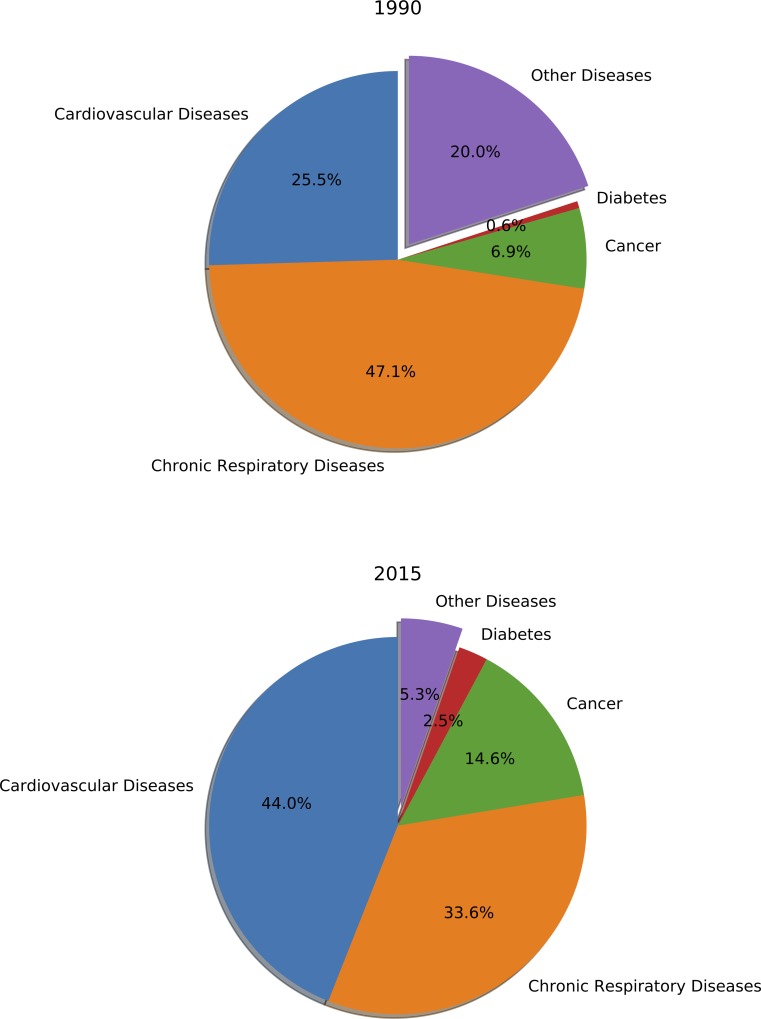
Percentage of deaths caused by air pollution by disease categories.

Fig 2 examines deaths associated with air pollution by disease. In 1990, 80.0% of air pollution–related deaths were from cardiovascular diseases, chronic respiratory diseases, cancer, and diabetes (the remaining air pollution–related deaths were from communicable diseases such as lower respiratory infections). This number jumped to 94.7% in 2015.

Among these four diseases, cardiovascular diseases and chronic respiratory diseases predominate, followed by cancer and diabetes. However, over the past decades, chronic respiratory disease mortality has decreased, while cardiovascular disease, cancer, and diabetes mortality have increased.

**Fig 3:**
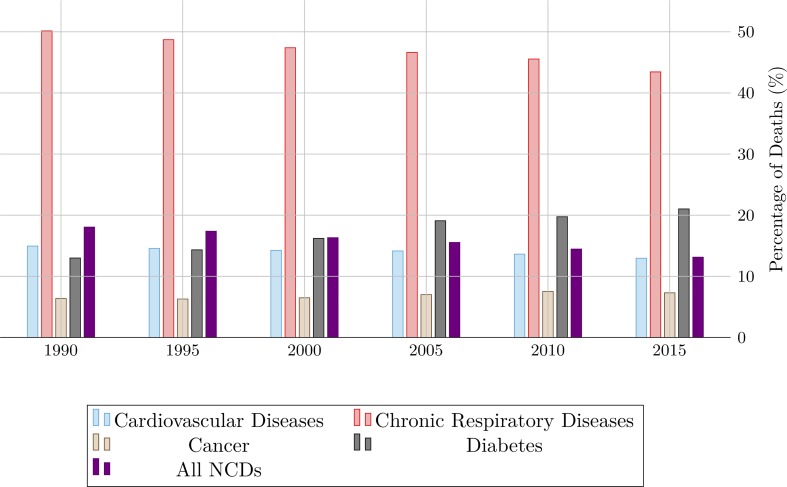
Percentage of deaths associated with air pollution for four disease categories, 1990–2015.

Fig 3 shows the proportion of deaths associated with air pollution for four disease categories in 1990–2015. Air pollution alone is linked to 18.1% of all NCD deaths in 1990. However, the proportion of NCD deaths associated with air pollution decreased recently: in 2015, air pollution is linked to 13.1% of all NCD mortality. Air pollution is associated with a greater proportion of chronic respiratory deaths than any other disease category.

### Macroeconomic burden of NCDs associated with air pollution

Table 1 shows the estimates of lost GDP due to cardiovascular diseases, chronic respiratory diseases, cancer, and all NCDs associated with air pollution in China in 1990–2030 and 2015–2030, respectively (in billions of 2010 USD). The total economic burden of NCDs associated with air pollution is estimated to be $1,137 billion (2010 USD) in 1990–2030 and $499 billion (2010 USD) in 2015–2030. Cardiovascular diseases have the largest economic burden among these NCD categories, followed by chronic respiratory diseases, diabetes, and cancer (in both time ranges).

**Table 1:** Estimates of lost GDP due to the four leading NCDs and due to all NCDs associated with air pollution in China (in billions of 2010 USD).

Disease	Total disease burden (billions of 2010 USD)	
	1990–2030	2015–2030
Cardiovascular diseases	478	199
Chronic respiratory diseases	294	125
Cancer	121	53
Diabetes	243	122
All NCDs^a^	1,137	499

^a^All NCDs associated with air pollution include cardiovascular diseases, chronic respiratory diseases, cancer, and diabetes.

Table 2 provides the estimates for the total disease burden due to the four NCDs (and all NCDs) associated with air pollution in China in 1990–2030 or 2015–2030, compared with total GDP in 2010 and divided by the population to frame the extent of the problem. These estimates show that the total disease burden associated with air pollution as a percentage of aggregate GDP in 2010 is 18.6% for the period 1990–2030 and 8.2% for the period 2015–2030. The per capita figures amount to a loss of $856 (in constant 2010 USD) and $322 in 1990–2030 and 2015–2030, respectively. If we relate the total NCD burden for China to the total output for the period 1990–2030 and adjust it for the projected growth rate, the burden of NCDs associated with air pollution is equivalent to an annual tax of about 0.36% on aggregate income in China.

**Table 2:** Economic burden due to the four NCDs and all NCDs associated with air pollution in China in 1990–2030 and 2015–2030 for different measures of economic performance.

Disease	1990–2030			2015–2030		
	% of 2010 GDP	Per capita loss (2010 USD)	% of total GDP in 1990–2030	% of 2010 GDP	Per capita loss (2010 USD)	% of total GDP in 2015–2030
Cardiovascular diseases	7.8%	360	0.15%	3.3%	142	0.09%
Chronic respiratory diseases	4.8%	222	0.09%	2.0%	89	0.05%
Cancer	2.0%	91	0.04%	0.9%	38	0.02%
Diabetes	4.0%	183	0.08%	2.0%	87	0.05
All NCDs^a^	18.6%	856	0.36%	8.2%	322	0.21%

^a^All NCDs associated with air pollution include cardiovascular diseases, chronic respiratory diseases, cancer, and diabetes.

We also explore the contribution of the treatment cost effect to our estimates of the total disease burden associated with air pollution, as is shown in Table 3. The treatment cost effect is calculated as the output difference between the scenario where we consider all effects and the scenario where we only consider the mortality and morbidity impact (but not the impact of treatment costs). In China, treatment costs account for 28% of the total economic burden of NCDs associated with air pollution. When we compare across diseases, the treatment cost effect is highest for diabetes, followed by chronic respiratory diseases, cardiovascular diseases, and cancer. These estimates show that treatment costs drive the total burden of diabetes associated with air pollution (i.e., 50.6%) to a much larger extent than they do for cancer (i.e., 9.9%).

**Table 3:** Economic burden due to the four NCDs and all NCDs associated with air pollution, excluding treatment cost effect in China, 1990–2030.

Disease	Total disease burden if we do not consider the impact of treatment cost (billions of 2010 USD)^a^	Treatment cost effect^b^
Cardiovascular diseases	414	13.4%
Chronic respiratory diseases	180	38.8%
Cancer	109	9.9%
Diabetes	120	50.6%
All NCDs^c^	822	27.7%

^a^If we only consider the effects of mortality and morbidity.

^b^Treatment cost effect = the percentage of the total economic burden of NCDs attributable to treatment cost.

^c^All NCDs associated with air pollution include cardiovascular diseases, chronic respiratory diseases, cancer, and diabetes.

## Discussion

Our analysis estimates that the macroeconomic losses of NCDs associated with air pollution are approximately $1,137 billion in 1990–2030, equivalent to roughly a 0.4% annual reduction in the country’s productive capacity. This loss is equivalent to 19% of China’s 2010 GDP, or $856 on a per capita basis. More than 27% of the total NCD burden associated with air pollution comes from high treatment costs, especially from diabetes and chronic respiratory diseases. Air pollution impeded China’s economic development in 1990–2015 and will continue to slow economic development if the status quo continues, for the macroeconomic losses of NCDs associated with air pollution are estimated to be approximately $499 billion in 2015–2030.

The Chinese government has pledged to accelerate efforts to improve air quality and has made some progress. For example, China has been working on reducing and banning the use of beehive coke ovens, which can emit many air pollutants, including carcinogenic polycyclic aromatic hydrocarbons that are linked to cancer. A recent study showed that since China legally banned beehive coke ovens in 1996, significant health benefits have been gained through the reduction of lung cancer [57]. China also issued the Air Pollution Prevention and Control Action Plan in 2013 to curb pollution, and noteworthy air quality improvements have occurred in many cities [58], with a 33.3% decline in PM2.5 concentrations and a 27.8% decline in PM10 concentrations. As a result, this policy averted approximately 50,000 deaths [58]. Notwithstanding this progress, challenges remain. For example, more cars are on the road, increasing hazardous emissions. According to the Traffic Management Bureau of China, the number of vehicles registered has increased from less than one million in 1990 to 300 million in 2017 [59].

In addition to promoting new climate policies such as limiting emissions, reducing the use of fossil fuels, and developing renewable energy, China could benefit tremendously from targeted public health policies. Preventive interventions could reduce the macroeconomic losses due to NCDs associated with air pollution: in particular, by encouraging people to wear masks on smoggy days; by using air purifiers in homes, offices, and cars; and by administering regular medical examinations for workers. All of these interventions could contribute to China’s economic growth by lowering the burden of NCDs associated with air pollution.

The data also show disparities in the disease-specific burden associated with air pollution. Cardiovascular diseases impose the largest macroeconomic loss, estimated to be $478 billion in 1990–2030, followed by chronic respiratory diseases, diabetes, and cancer. Because resources are limited, we must prioritize and allocate resources efficiently. Different interventions may target different populations and, therefore, have different consequences on different diseases. Maximizing resources may mean investing in air pollution control initiatives that target the working-age population and result in greater reduction in cardiovascular diseases, which may be more cost-beneficial than targeting older cohorts or focusing on other diseases.

This study accounts for several influences of NCDs associated with air pollution on economic growth: mortality, morbidity, and treatment cost effects. We also incorporate age-specific human capital to account for education-related productivity differences among members of different cohorts who are differentially affected by NCDs. While this allows for a comprehensive analysis of the macroeconomic burden of NCDs, the framework does not yet allow for feedback effects between diseases and research and development expenditures to account for endogenous long-run growth [60, 61]. Note also that our study’s results likely underestimate the total impact of air pollution on the Chinese economy, as our estimates only address the economic burden of NCDs associated with air pollution. In reality, air pollution is also closely associated with acute respiratory diseases; in China, 35% of deaths due to lower respiratory infections in 2017 were associated with air pollution [62]. Exposure to air pollution may also impede cognitive performance, brain imaging, and dementia [63–65]. Furthermore, air pollution also affects aggregate production output through other channels such as crop loss, forest damages, and material damages [66, 67], which we do not account for.

## Supporting information

S1 Appendix Mathematical formulation and data sources. (DOCX)
