# Supplementary material for: The macroeconomic burden of noncommunicable diseases associated with air pollution in China
Source: PLoS One. 2019 Apr 18;14(4):e0215663. doi: 10.1371/journal.pone.0215663 (PMC6472813; doi:10.1371/journal.pone.0215663)
Supplement: S1 Appendix — (DOCX) [file pone.0215663.s001.docx]

**S1 Appendix: Mathematical formulation and data sources**

In this S1 Appendix to the PLOS ONE paper “The macroeconomic burden of noncommunicable diseases associated with air pollution in China,” we provide the mathematical formulation of our model and information on the data sources.

**A. Mathematical formulation**

**Modeling details**

In this analysis, we quantify its impact on economic outputs through the increasing prevalence of noncommunicable diseases. For each disease, we first identify the disease burden associated with air pollution (in terms of mortality, morbidity, and treatment cost). Then we construct economic projections for the two scenarios of interest following the model in [1]. The economic loss is calculated as the undiscounted cumulative difference in projected annual GDP between these two scenarios. The following detailed description of the model follows our previous contributions in which we applied the framework to China, Japan, and South Korea [1] and to the United States [2].

**Production function**

Consider an economy in which time $t\in\left[ 0, \infty\right]$ evolves discretely. Building upon Lucas [3], we consider the following production function for the Chinese economy:

$$\begin{aligned} Y_{t}=A_{t}K_{t}^{\alpha}H_{t}^{1-\alpha}\#\left( 1 \right) \end{aligned}$$

where $A_{t}$ is the technological level at time $t$ that we assume evolves exogenously, $K_{t}$ is the physical capital stock (i.e., machines, factory buildings, etc.), and $H_{t}$ represents aggregate human capital. The parameter $\alpha$ is the elasticity of final output with respect to physical capital. The aggregate production function takes into account that output is not only produced with physical capital and *raw labor,* as in the Solow framework [4] on which the original model relies, but also with *effective labor* for which health is a crucial determinant.

Physical capital evolves according to

$$\begin{aligned} K_{t+1}=\left( 1-\delta\right)K_{t}+Y_{t}-C_{t}-\sum_{j\in I} TC_{j,t}=\left( 1-\delta\right)K_{t}+s_{t}Y_{t}\#\left( 2 \right) \end{aligned}$$

where $\delta$ is the rate of depreciation and $s_{t}$ refers to the savings rate. From Equation (2), it follows that the savings rate is defined as

$s_{t}=1-\frac{C_{t}+\sum_{j\in I} TC_{j,t}}{Y_{t}}$.

Note that aggregate output $Y_{t}$ is used for three purposes: 1) to pay treatment costs $TC_{j,t}$ (these costs refer to the costs of ongoing treatment and of the intervention methods for the specific disease; in general, these costs are best characterized by the direct costs of health care, including hospitalization, medication, etc.) for disease $j\in I$, where $I$ is the set of diseases that we consider; 2) to consume the amount $C_{t}$; and 3) to save. In other words, we assume that the treatment costs do not contribute to physical capital accumulation. Hence, the real loss associated with treatment costs only amounts on the drag imposed on savings and thus capital accumulation.

Individuals of age group $a$ are endowed with $h_{t}^{(a)}$ units of human capital and supply $\mathcal{l}_{t}^{(a)}$ units of labor from age 15 up to their retirement at age $R$, i.e., for $a\in[15, R]$. Children below the age of 15 and retirees above the age of $R$ do not work. Actually, *R* varies by country and could be a very high number (e.g., some people aged above 80 could also be working). *R* just indicates the upper bound of the summation equation. In our analysis, we use the labor projections data from the International Labour Organization, and a positive labor force exists for the age cohort 65 years and older. Aggregate human capital in the production function (1) is then defined as the sum over the age-specific effective labor supply of each age group:

$$\begin{aligned} H_{t}=\sum_{a=15}^{R} h_{t}^{\left( a \right)}\mathcal{l}_{t}^{\left( a \right)}N_{t}^{\left( a \right)}\#\left( 3 \right) \end{aligned}$$

where $N_{t}^{a}$ denotes the number of individuals belonging to age group $a$. Note that aggregate human capital increases with the number of working-age individuals in the economy (i.e., with a higher $N_{t}=\sum_{a=15}^{R} N_{t}^{(a)}$ is larger), with individual human capital endowment (i.e., with a higher $h_{t}^{(a)}$ for at least one $a$), and with labor supply (i.e., with a higher $\mathcal{l}_{t}^{(a)}$ for at least one $a$).

We follow Mincer [5] and construct average human capital of the cohort aged $a$ according to an exponential function of education and work experience:

$$\begin{aligned} h_{t}^{\left( a \right)}=\exp\left[ \eta_{1}\left( ys_{t}^{\left( a \right)} \right)+\eta_{2}\left( a-ys_{t}^{\left( a \right)}-5 \right)+\eta_{3}\left( a-ys_{t}^{\left( a \right)}-5 \right)^{2} \right],\#\left( 4 \right) \end{aligned}$$

where $\eta_{1}$ is the semi-elasticity of human capital with respect to average years of education as given by $ys_{t}^{\left( a \right)}$, and $\eta_{2}$ and $\eta_{3}$ are the semi-elasticities of human capital with respect to the experience of the workforce $\left( a-ys_{t}^{\left( a \right)}-5 \right)$ and the experience of the workforce squared $\left( a-ys_{t}^{\left( a \right)}-5 \right)^{2}$, respectively.

**Impact of NCDs associated with air pollution**

Air pollution’s associated mortality (and morbidity) of NCDs is (are) expected to reduce effective labor supply in the economy. In other words, by reducing NCDs’ prevalence, the *counterfactual scenario* is associated with an increase in labor supply as compared with the *status quo scenario*. Following [1], we approximate the change in labor supply (at time $t$ for age group $a$) by

$$\begin{aligned} \Delta L_{t}^{\left( a \right)}\approx\mathcal{l}_{t}^{\left( a \right)}N_{t}^{\left( a \right)}\sum_{\tau=0}^{\min\left\{ t,a \right\}-1} \sigma_{i,t-1-\tau}^{\left( a-1-\tau\right)}\left[ 1+p_{i}^{\tau}\xi_{i}^{\left( a-1-\tau\right)} \right]\#\left( 5 \right) \end{aligned}$$

where $\sigma_{i,t}^{\left( a \right)}$ is the mortality effect of disease $i$ over age group $a$ at time $t$, $\xi_{i}^{\left( a \right)}$ measures the relative size of the morbidity effect, and $p_{i}^{t}$ is the probability that a sick person fails to recover from the disease until time $t$.

Because the impact of morbidity is hard to estimate directly, we first define

$$\begin{aligned} \xi_{i}^{\left( a \right)}=\frac{loss of labor due to morbidity in age group a}{loss of labor due to mortality in age group a}\#\left( 6 \right) \end{aligned}$$

Next, we assume that the following holds in any given year for each age group $a$:

$$\begin{aligned} \xi_{i}^{\left( a \right)}=\frac{YLD_{i}^{\left( a \right)}}{YLL_{i}^{\left( a \right)}}\#\left( 7 \right) \end{aligned}$$

where $YLD_{i}^{\left( a \right)}$ represents the years lived with disease $i$ and $YLL_{i}^{\left( a \right)}$ represents the years of life lost due to disease $i$. Notice that $\xi_{i}^{\left( a \right)}$ can be calculated from the corresponding disability-adjusted life year data reported by the Global Burden of Disease Study [6]. The disease burden of NCDs also impedes the accumulation of physical capital in the sense that savings finance part of the treatment costs. Following [1], physical capital accumulation in the counterfactual scenario can be written as

$$\begin{aligned} \bar{K}_{t+1}=\bar{s}_{t}\bar{Y}_{t}+\left( 1-\delta\right)\bar{K}_{t}\#\left( 8 \right) \end{aligned}$$

$$\bar{s}_{t}\bar{Y}_{t}=\bar{I}_{t}=\bar{Y}_{t}-\bar{C}_{t}-\sum_{j\in I,j\neq i} TC_{j,t}=s_{t}\bar{Y}_{t}+\chi TC_{i,t}$$

where $\bar{s}_{t}$ is the savings rate in the counterfactual scenario. This savings rate is in turn defined by

$$\bar{s}_{t}=\frac{s_{t}\bar{Y}_{t}+\chi TC_{i,t}}{\bar{Y}_{t}}$$

where $\chi$ is the fraction of the treatment cost that is diverted to savings and $TC_{i,t}$ is the treatment cost for disease $i$ at time $t$.

**B. Data sources**

Table A shows the estimates of treatment costs for NCDs associated with air pollution in China as of 2015 (in constant 2010 USD).

For diabetes, we obtained the treatment cost information for China from the International Diabetes Federation [7].

For the other three disease categories, to our best knowledge, no treatment cost data for China is available. Thus, we sourced the treatment cost data from other countries.

First of all, we extract national disease-specific treatment cost for other countries available.

- For cancer, we get the treatment cost data for 27 European Union countries from [8], and United States from [9].
- For cardiovascular diseases, we get the treatment cost data for 27 European Union countries from [10], and United States from [9].
- For chronic respiratory disease, we get the treatment cost data for US from [9].

Second, for each disease category, we then calculate the per case costs for the countries with data and extrapolated costs for China under the assumption that the per case costs are proportional to the per capita health expenditure (from World Bank [11]). This technique has been proposed and used in [12] and [13].

Now we have the disease-specific treatment cost per capita (transformed from per case costs with prevalence data from GBD). We then approximate the treatment cost associated with pollution by assuming that the cost is proportional to DALYs (the data for DALY are from GBD 2017 [6]). For example, if air pollution accounts for 7% of the total DALYs of cancer according to GBD 2017, the respective treatment cost associated with air pollution for cancer will then be calculated as 0.07*total treatment cost of cancer.

Treatment costs used for other years are scaled according to the projection of health expenditure per capita.

**Table A. Estimates of treatment cost of NCDs associated with air pollution in China (in 2010 USD)**

| Disease | Treatment cost per capita (in 2010 USD) |
| --- | --- |
| Cardiovascular disease | 2.48 |
| Chronic respiratory disease | 4.39 |
| Cancer | 0.45 |
| Diabetes | 4.73 |

Table B shows the parameter values we employ for our numerical analysis. Note that we take the coefficient (the output elasticity of physical capital) from Penn World Table 2014 [14]. Penn World Table 2014 [14] also starts from a Lucas Growth Model and approximates the output elasticity of capital with the capital share of GDP for a range of countries including China.

**Table B. Parameter values and data sources**

| Parameter | Value | Source |
| --- | --- | --- |
| $\alpha$ | 0.43 | [14, 15] |
| $\delta$ | 0.05 | [16] |
| $s$ | 48.7% | [17] |
| $\eta_{1}$ | 0.102 | [18] |
| $\eta_{2}$ | 0.1301 | [19] |
| $\eta_{3}$ | −0.0023 | [19] |
| $\chi_{i}$ | Set as savings rate | [17] |

**References**

1. Bloom DE, Chen S, Kuhn M, McGovern ME, Oxley L, Prettner K. The economic burden of chronic diseases: estimates and projections for China, Japan, and South Korea. The Journal of the Economics of Ageing. 2018. doi: <https://doi.org/10.1016/j.jeoa.2018.09.002>.

2. Chen S, Kuhn M, Prettner K, Bloom DE. The macroeconomic burden of noncommunicable diseases in the United States: Estimates and projections. PLoS One. 2018;13(11):e0206702. doi: 10.1371/journal.pone.0206702.

3. Lucas RE. On the mechanics of economic development. J Monet Econ. 1988;22(1):3-42.

4. Solow RM. A contribution to the theory of economic growth. The Quarterly Journal of Economics. 1956;70(1):65–94.

5. Mincer J. Schooling, experience, and earnings. Human Behavior & Social Institutions No. 2. 261 Madison Ave., New York, New York 10016: National Bureau of Economic Research Inc.; 1974.

6. James SL, Abate D, Abate KH, Abay SM, Abbafati C, Abbasi N, et al. Global, regional, and national incidence, prevalence, and years lived with disability for 354 diseases and injuries for 195 countries and territories, 1990–2017: a systematic analysis for the Global Burden of Disease Study 2017. The Lancet. 2018;392(10159):1789-858.

7. International Diabetes Federation. IDF Diabetes Atlas 7th Edition. Brussels, Belgium: International Diabetes Federation, 2015.

8. Luengo-Fernandez R, Leal J, Gray A, Sullivan R. Economic burden of cancer across the European Union: a population-based cost analysis. The lancet oncology. 2013;14(12):1165-74.

9. Dieleman JL, Baral R, Birger M, Bui AL, Bulchis A, Chapin A, et al. US spending on personal health care and public health, 1996–2013. JAMA. 2016;316(24):2627–46.

10. Wilkins E, Wilson L, Wickramasinghe K, Bhatnagar P, Leal J, Luengo-Fernandez R, et al. European cardiovascular disease statistics 2017. 2017.

11. World Bank. World Bank database, current health expenditure (% of GDP) Washington, DC: World Bank; 2017 [cited 2018 April 20]. Available from: <https://data.worldbank.org/indicator/SH.XPD.CHEX.GD.ZS>.

12. Bloom DE, Cafiero E, Jané-Llopis E, Abrahams-Gessel S, Bloom L, Fathima S, et al. The global economic burden of noncommunicable diseases. Geneva, Switzerland: World Economic Forum, 2011.

13. Ding D, Lawson KD, Kolbe-Alexander TL, Finkelstein EA, Katzmarzyk PT, van Mechelen W, et al. The economic burden of physical inactivity: a global analysis of major non-communicable diseases. The Lancet. 2016;388(10051):1311-24.

14. Feenstra RC, Inklaar R, Timmer MP. The next generation of the Penn World Table. Amer Econ Rev. 2015;105(10):3150-82.

15. Jones CI. R&D-based models of economic growth. J Polit Economy. 1995;103(4):759-84.

16. Grossmann V, Steger T, Trimborn T. Dynamically optimal R&D subsidization. J Econ Dynam Control. 2013;37(3):516-34.

17. World Bank. World Bank database, gross savings (% of GDP) 2018 [cited 2018 March 20]. Available from: <https://data.worldbank.org/indicator/NY.GNS.ICTR.ZS>.

18. Psacharopoulos G, Patrinos HA. Returns to investment in education: a decennial review of the global literature. Educ Econ. 2018:1-14.

19. Heckman JJ, Lochner LJ, Todd PE. Earnings functions, rates of return and treatment effects: The Mincer equation and beyond. Handbook of the Economics of Education. 2006;1(2006):307–458. doi: 10.1016/S1574-0692(06)01007-5.
